# Lymphangioplasty

**Published:** 1908-12-12

**Authors:** 


### SPECIAL ARTICLE.

### LYMPHANGIOPLASTY.

One of the major complications of carcinoma mammæ is the brawny swelling of the arm that comes on in the later stages of the disease in a certain number of cases. The excruciating pain that results is worse than that of the cancer itself; so much so that the limb has even had to be amputated to afford some slight relief. It is to be hoped that a day will come when the primary growths will be amenable to cure, so that cases of brawny arm swelling will not occur. Meanwhile, however, something has to be done to deal practically with the sufferers, and the operation of lymphangioplasty promises to go a long way towards the relief of that which has hitherto been regarded as almost hopeless. An excellent account of the *raison d'être* of the operation, and of the results obtainable by it, will be found in the thirteenth volume of the Archives of the Middlesex Hospital, in a paper by Mr. W. Sampson Handley, who devised the operation in question. The procedure consists in providing channels along which lymph may pass from the distal end of the limb to the patient's trunk, by running continuous buried silk strands along up the arm, across the armpit, and down the chest wall. These silk strands act like wicks, and enable the lymph to pass across the site of the lymphatic obstruction in the axilla, and there is no doubt whatever of the enormous relief that the operation has afforded to some of the patients who have been under Mr. Handley's care.

Mr. Handley tells us that the brawny arm occurs in about one case in every six of breast cancer. It was formerly considered to be due to pressure of a mass of secondary growth upon the axillary veins; this view is still adopted in some books. There is abundant evidence however to show that venous obstruction is not the cause of brawny arm. During operation the axillary vein has often been ligatured, without any œdema resulting, and brawny arm often occurs in cases which present no mass of axillary growth, but is rather associated with atrophic forms of the disease.

If lymphatic obstruction is the cause of the brawny arm, how does this obstruction come about? Is it by the interior of the lymphatics becoming blocked by carcinoma cells? If so, do these cells reach the lymphatics by a process of embolism, or by a continuous infiltration from the primary cancer? If the latter is the case, how far along the lymphatics of a brawny arm does actual cancer extend? Is there any accessory process taking place in the lymphatics in addition to infiltration by carcinoma? If so, what is its precise nature?

All these questions needed a precise answer before the operation of lymphangioplasty could be devised. The following is Dr. Handley's account of what is termed the *permeation theory* of the spread of cancer by lymphatics. The cancer cells spread centrifugally in all directions from their point of origin by actually growing along and filling up the lymphatic plexus to which they have obtained access. This process of permeation may slowly spread in a circle any distance up to about two feet from its point of origin. The cancer cells would not by themselves absolutely obstruct the lymphatic vessels, but a permeated lymphatic is ultimately doomed to destruction by a process of peri-lymphatic fibrosis which, though induced by the presence of cancer, is not itself malignant, but rather a reparative process. It takes place as follows:—The plug of cancer cells within the lymphatic continuing to proliferate finally splits up the lymphatic. Around the microscopic injury thus produced a vigorous round-celled infiltration occurs, to be replaced later by a capsule of new-formed fibrous tissue which contracts upon and ultimately strangles the enclosed cylinder of cancer cells. The original lymphatic vessel becomes replaced by a solid fibrous cord. The recognition of the fact that the permeative spread of a cancer is accompanied by an almost co-extensive destruction of the lymph-vascular system by fibrosis for the first time affords an adequate pathology for the swollen arm of breast cancer. The circle of permeation, spreading from the breast, sooner or later reaches the arm; after it has involved the whole circumference of the latter, and has been succeeded by the obliterative process of peri-lymphatic fibrosis, the arm becomes entirely cut off from the lymph-vascular system of the rest of the body. It is a very important part of this view of the pathology of the condition that although cancerous deposits start the obstruction, a great many of the fibrosed lymphatics may ultimately exhibit no cancer cells at all.

Obviously the limb requires a new set of lymphatics. Mr. Handley has tried to supply a substitute by means of wicks. The next point he had to decide was what to use for these; it had to be something along which fluids would pass by capillary attraction, yet something that would not become blocked by being penetrated by leucocytes, connective tissue, or cancer cells. Silk suggested itself, and it became of extreme importance to know whether silk did or did not become permeated by tissue cells when it remained long embedded in the body. The answer to this point in pathology was obtained from sections of a silk ligature that had been ten years in the peritoneum. Though eroded at its surface, the main substance of the thread was intact. Moreover, leucocytes are evidently unable to penetrate between the fibrils of the silk, for the section showed the interior of the thread to be entirely free from cellular elements; nor was there any microscopic indication of the coagulation of the body fluids in its interstices. It appeared therefore that a stout silk thread would retain its capillary power and remain unabsorbed if it were introduced subcutaneously into the arm, running longitudinally upwards from the wrist to terminate in the loose and healthy areolar tissue on the proximal side of the axilla. The results of introducing a series of these wicks in apparently hopeless cases of brawny arm have even exceeded the hopes that were entertained. The operation is styled lymphangioplasty.

